# A bibliometric and visual analysis of low carbohydrate diet

**DOI:** 10.3389/fnut.2023.1085623

**Published:** 2023-02-23

**Authors:** Gang Lu, Xin Huang, Chun Lin, Lijuan Zou, Huashan Pan

**Affiliations:** ^1^Clinical Medical College of Acupuncture Moxibustion and Rehabilitation, Guangzhou University of Chinese Medicine, Guangzhou, China; ^2^School of Physical Education and Health, Guangzhou University of Chinese Medicine, Guangzhou, China; ^3^Science and Technology Division, Guangdong Food and Drug Vocational College, Guangzhou, China

**Keywords:** bibliometric analysis, low carbohydrate diet, CiteSpace, VOSviewer, hotspots, frontiers trends

## Abstract

**Introduction:**

Numerous studies have confirmed the effects of low carbohydrate diet (LChD) on metabolism and chronic diseases. However, there were no bibliometric studies on LChD. This study was conducted through a bibliometric analysis to investigate the current status, hotspots and frontiers trends.

**Methods:**

We searched all research publications related to LChD from 2002 to 2021 on the Web of Scientific Core Collection (WoSCC). CiteSpace and VOSviewer software was used to analyze countries/regions, institutions, journals, authors, references, and keywords.

**Results:**

A total of 6938 papers were included, with an increasing trend of annual publication. LChD categories mainly included nutrition, endocrinology, and neurosciences which reflected the interdisciplinary characteristics. USA was with the largest number and the world science center in LChD field. Universities were main research institutions and five of the top 10 institutions were from USA. Eric Heath Kossoff had 101 publications and ranked first. Nutrients was the leading journal. “A randomized trial of a low-carbohydrate diet for obesity” and “Obesity” were considered to be the most co-cited and cited reference respectively. The hotspots of LChD are four aspects, “ketogenic diet”, “metabolism disease”, “cardiovascular disease” and “cancer”. We summarized that “oxidative stress”, “gut microbiota”, and “inflammation factors” are becoming frontiers trends of LChD research in the future and deserve further study.

**Discussion:**

Over the past 20 years research on LChD has gained great attention. To better explore LChD field, multilevel mechanism studies will be required in the future.

## Introduction

Obesity-related complications affect almost all body systems and are significant risk factors for coronary heart disease, type 2 diabetes, cancers such as endometrial, breast, prostate, and skin cancers, as well as several other chronic non-communicable diseases. Diet therapy methods, theories, and applications are constantly updated as a result of ongoing research on the metabolism of the organism in normal and disease states (1). It has been demonstrated that consuming a diet high in carbohydrate increases the risk of developing metabolic and chronic diseases, and that lowering carbohydrate intake decreases the incidence of morbidity (2). The effects of LChD on health have garnered a lot of attention recently. There are many types of low carbon diet prescriptions according on the carbohydrate intake ratio. The American Diabetes Association recommended a conventional 2,000 calorie daily diet with < 130 g of carbohydrates (3). The other study suggested consuming < 40% of one's daily calories from carbohydrates (4). Anyway, the two prescriptions LChD above are powered by glucose first and then switch to ketone bodies after fasting. Moreover, the ketogenic diet (KD) is another LChD that calls for a very low carbohydrate intake (< 10%). KD, a sort of LChD, was initially used to cure epilepsy (5). Atkins, an American, wrote about an LChD in his 1972 book “*Dr. Atkins' New Diet Revolution*,” in which the intake of carbohydrate was rigorously limited while the intake of protein and fat is raised (6). Currently modified Atkins diet, a easier KD, has showed very similar effects with KD (7). A LChD can lower excess body weight (8, 9), as well as the risk of diabetes, cancer, cardiovascular disease, and internal inflammatory responses brought on by obesity (10, 11). Of fact, some research has indicated that LChD can also produce negative health effects, such as gastric dysfunction (12), atherosclerosis (13), physical fatigue (14), etc.

Studies on LChD are becoming increasingly popular in recent years as a response of the academic community's intense interest in the disease's favorable health effects (15–17). Most of studies, nevertheless, have concentrated on how LChD affects certain disease locations. We require a thorough understanding of the development process and research trends in this subject given the rapid proliferation of research on LChD. However, there are no bibliometric and visual analysis article on LChD.

Bibliometrics, a mathematical and statistical tool for quantitatively analyzing all knowledge (18), has been used to assess distributions, collaboration, citation, keywords, hotspots, and frontiers trends (19). CiteSpace and VOSviewer are software for visualization for bibliometrics analysis (20, 21). These two software generate network maps that allow researchers to intuitively analyze the current status within the field, and determine the research hotspots and frontiers trends (22). Therefore, this study employs CiteSpace and VOSviewer software to analyze the publications on LChD from 2002 to 2021, to evaluate and analysis the research hotspots and frontiers trends. This has been the first study to use bibliometric strategies in the field of LChD. The study is expected to help researchers extract potential information for further research in the field of LChD research and offer them helpful advice in choosing ground-breaking subject matter by answering the following questions:

- Which countries, institutions, journals, authors, and references are the current status of research in the field of LChD ?
- What are the current hotspots and major categories of LChD?
- Where are in the future frontiers trends of LChD ?

## Materials and methods

### Data acquisition and search strategy

In this study, WoSCC was selected as the data source. As a high-quality digital literature resource database, WoSCC has been accepted by many researchers, and considered as the most suitable database for literature analysis (23). All publications were retrieved from the Science Citation Index Expanded (SCI-E) of the WoSCC database on November 12, 2022. We completed the search within the same day to avoid any bias caused by database updates. The following methods were conducted for search publications: topic words = (“low carbohydrate” OR “low-carbohydrate” OR “low carb” OR “low-carb” OR “ketogenic” OR “carbohydrate-restricted” OR “carbohydrate restricted” OR “restricted carbohydrate” OR “restricting carbohydrate” OR “carbohydrate restriction” OR “South Beach diet” OR “Atkins diet”). In order to more accurately analyze the current status, hotspots and frontiers trends of LChD, the publications from 2002 to 2021 were selected. Time span = January 1, 2002–December 31, 2021. To ensure the representativeness of the included studies, the types of publications were limited to “articles” and “reviews” (24). No languages limitation to avoid bias in the geographical distribution of publications. The content of literature records were “full records and cited references,” downloaded and saved in plain text document format.

### Statistical analysis

We used the CiteSpace (6.1.R3) and VOSviewer (1.6.18) for a bibliometric analysis of 6,938 publications on LChD from 2002 to 2021. The java-based program CiteSpace does bibliometric analysis of publications using distribution network maps, co-citation network maps, dual maps of journal overlay, and keyword burst citation maps (25). Nodes and links are included in the visual network diagram produced by CiteSpace. Every node is a factor, such as an author, an institution, or a country (26). Links between different nodes show a network of relationships involving co-operation, co-citation, or co-occurrence (27) A wider line indicates a more effective collaboration. The higher the centrality, the larger the circle is in terms of centrality. When a node has a purple circle around it, it has a high centrality score and is therefore an important node in the field (22). VOSviewer was used to form keyword co-occurrence of overlay visualization. The colors represent the years (28). The size of the node is proportional to the frequency of keyword occurrences (29). Data was managed, charts were made, and all data tables were created using Microsoft Excel 2021 software.

## Results

### Annual output and categories

**Figure 1:**
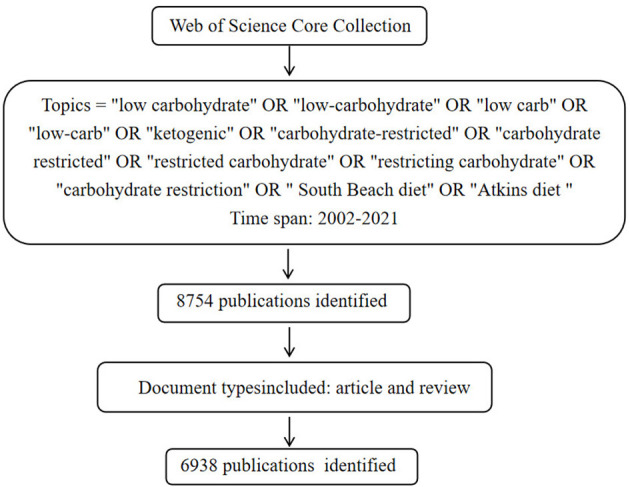
The flowchart searching papers in databases.

**Figure 2:**
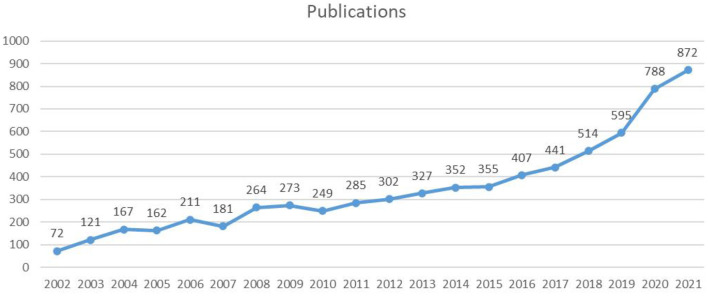
The annual number of publications on LChD from 2002 to 2021.

A total of 6,938 publications including 5,350 articles and 1,588 reviews, related to LChD from 2002 to 2021 were retrieved by searching the WoSCC database. The flowchart was shown in Figure 1. The annual publications reflected the activities in the field and the attention given to certain areas of research (30). As seen in Figure 2, the number of annual publications on LChD showed an overall upward trend in spite of fluctuation slightly in some years over the past 20 years. It indicates that LChD research is becoming a research of great interest to scholars and has attracted great interest from scholars in recent years.

LChD publications in the past 20 years can be divided into 2 stages. The initial stage (2002–2010) was a steady growth period. The average number of publications was 188 publications every year, with the lowest number of publications being 72 publications in 2002 and the highest number being 273 publications in 2009. In 1927, a low carbohydrate ketogenic diet had been reported for epilepsy (31). As an early study in 1948, LChD was used to control of dental caries (32). Since 2002, LChD was contributed to a variety of areas, including obesity (33), diabetes (34), and cardiovascular disease (35). Although the number of papers varied at this stage, the overall trend was one of consistent growth. The second stage (2011–2021) was a sustained growth period. The average number of publications annually was 476 publications. The number of publications reached 872 in 2021. Nutrition has a significant role in daily life, and it is crucial for the advancement of social development to support research on diet and health. LChD research has gained popularity as a nutritional approach and is rapidly developing into a research hotspot.

The categories refer to the disciplines covered by the dissertation research. At top 10 categories (Table 1), Nutrition Dietetics had 1,621 publications and ranked first, followed by Clinical Neurology (1,269 publications), Endocrinology Metabolism (960 publications), Neurosciences (734 publications) and Pediatrics (472 publications). LChD research mainly covered the fields of nutrition, endocrinology, and neurosciences, reflecting the multidisciplinary nature and comprehensive knowledge.

**Table 1:** The top 10 categories on LChD from 2002 to 2021.

**Rank**	**Category**	**Publications**
1	Nutrition dietetics	1,621
2	Clinical neurology	1,269
3	Endocrinology metabolism	960
4	Neurosciences	734
5	Pediatrics	472
6	Biochemistry molecular biology	417
7	Medicine general internal	314
8	Medicine research experimental	282
9	Pharmacology pharmacy	269
10	Multidisciplinary sciences	227

### Analysis of countries/regions

**Figure 3:**
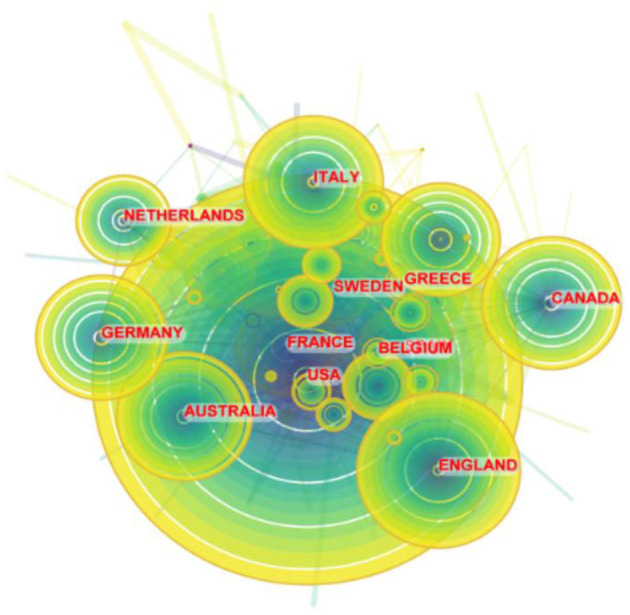
Map of countries/regions on LChD from 2002 to 2021.

In total, 112 countries/regions participated in 6,938 publications on LChD from 2002 to 2021. CiteSpace generated the countries/regions distribution map, and 112 nodes and 880 links were shown in the map (Figure 3). Table 2 presented the top 10 countries/regions published in LChD research field. USA had the highest number of publications, 2,862 papers, accounting for 41.25%. The Yuasa phenomenon states that the nation whose research output accounts for more than 25% of all scientific output at any given moment can be referred to as the world center of science during that time (36). As the leader in LChD research, USA published far more than a quarter of the total publications and was the world science center in the field of LChD. England (543 publications), Italy (472 publications), China (449 publications), and Germany (441 publications) followed closely behind. In terms of centrality, Canada (0.16) ranked first, followed by, Spain (0.14), Australia (0.13), England (0.13), France (0.11) and, which maintain close cooperation relationships. Countries/regions with centrality played an important role in LChD research. Germany, Canada, Australia and France each had < 450 publications, but their research roles were important. In terms of publications, China had 449 papers, but the centrality was only 0.01. It demonstrated that despite having a high publications number, China had few connections and little influence over the network map. The level of LChD research in China therefore was raised effectively by deepening the field's research, advancing cross-disciplinary and cross-field collaboration, and enhancing researchers' capacity for creative thinking and global communication.

**Table 2:** The top 10 countries/regions on LChD from 2002 to 2021.

**Rank**	**Country/region**	**Publications**	**Centrality**
1	USA	2,862	0.08
2	England	543	0.13
3	Italy	472	0.08
4	Germany	449	0.08
5	China	441	0.01
6	Canada	434	0.16
7	Australia	422	0.13
8	Japan	339	0.02
9	Spain	301	0.11
10	France	299	0.14

### Analysis of institutions

**Figure 4:**
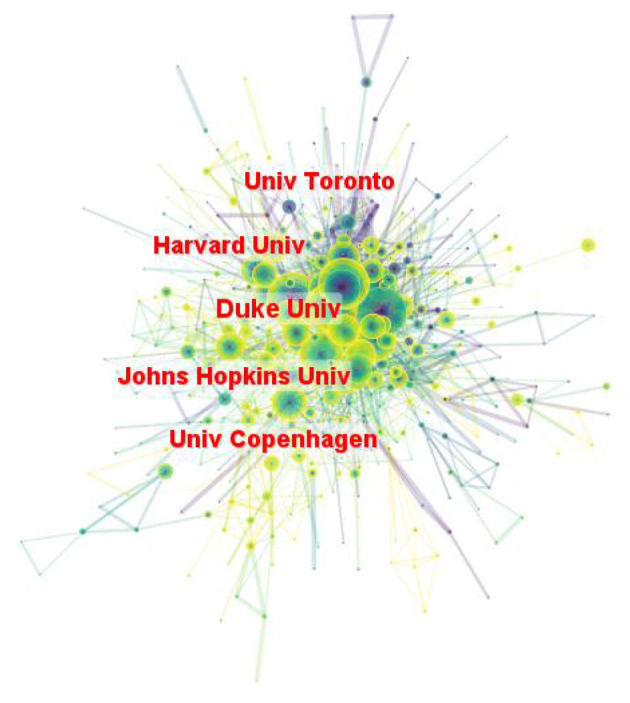
Map of institutions on LChD from 2002 to 2021.

A total of 604 institutions provided research in the field of LChD. CiteSpace generated the institutions distribution map with 604 nodes and 2,103 links (Figure 4). The institutions with large numbers of publications have been identified as influential institutions (37). Table 3 listed the top 10 institutions in publications, and they were the most influential institutions in LChD research. Universities were major institutions for LChD research. Harvard University ranking first, had 451 papers, followed by University of California System (258 publications), Johns Hopkins University (216 publications), Udice French research universities (191 publications), and University of London (174 publications). Five of the top 10 institutions were from USA, which further confirmed US predominance in the field of LChD research. Duke University, Harvard University, Johns Hopkins University and University of Toronto had close collaboration relationships.

**Table 3:** The top 10 institutions on LChD from 2002 to 2021.

**Rank**	**Institution**	**Country**	**Publications**
1	Harvard University	USA	451
2	University of California System	USA	258
3	Johns Hopkins University	USA	216
4	Udice, French research universities	France	191
5	University of London	England	174
6	Johns Hopkins Medicine	USA	146
7	Institut National de la Santé et de la Recherche Médicale	France	131
8	University of Toronto	Cananda	127
9	Assistance Publique Hopitaux Paris Aphp	France	125
10	University of Connecticut	USA	124

### Analysis of authors

**Figure 5:**
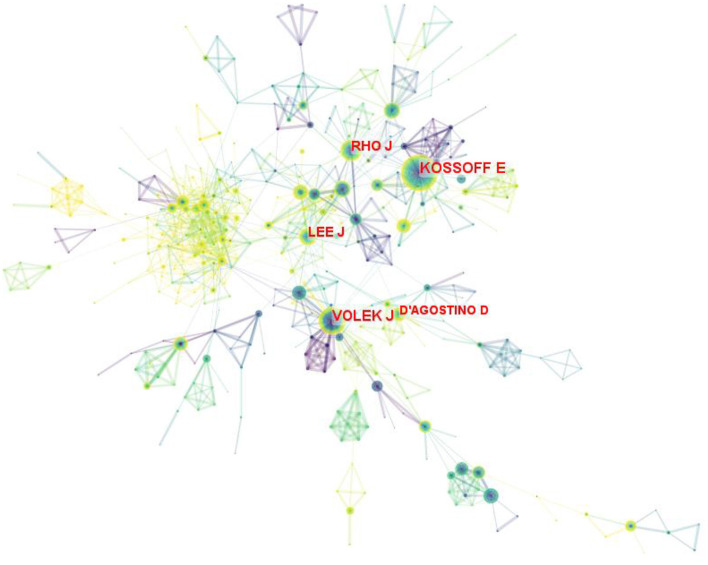
Map of authors on LChD from 2002 to 2021.

In total of 890 authors participated in 6,938 publications on LChD from 2002 to 2021. CiteSpace generated the institutions distribution map with 890 nodes and 1965 links (Figure 5). The top 10 authors participating in the LChD research are shown in Table 4. The most productive authors were Eric Heath Kossoff (101 publications), Jeff Scott Volek (69 publications), Jong M. Rho (62 publications), William S. Yancy (45 publications), and Maria Luz Fernandez (43 publications). Eric Heath Kossoff ranked first in the number of publications devoted to the study of the effects of a high-fat, low-carb ketogenic diet on neurological disorders. He demonstrated that a high-fat, low-carb ketogenic diet reduced the number of seizures in refractory epilepsy and reported no cardiovascular or cerebrovascular events (38, 39). In addition ketogenic diets are being applied to a range of neurological disorders from autism to Alzheimer's disease (40). Jeff Scott Volek was the second position of papers. He reported that in individuals with atherosclerotic dyslipidemia, a 12-week carbohydrate restriction diet improved postprandial vascular function more than a low-fat diet (41). An study revealed that LChD (10%) not only decreased lipid deposition but avoided the buildup of plasma and aortic oxidation, decreased inflammatory cytokines within the artery wall, and prevented atherosclerosis (42). Jong M. Rho was in the third place in terms of number of publications. In addition to a high-fat, low-carbon-water ketogenic diet that improves epilepsy (43), he emphasized that a ketogenic diet enhances mitochondrial function and reduces autistic behavior in humans and rodent models of autism spectrum disorder (44, 45). The authors' collaboration displayed a geographical concentration and general decentralization.

**Table 4:** The top 10 authors on LChD from 2002 to 2021.

**Rank**	**Author**	**Affiliations**	**Publications**
1	Kossoff Eric H.	Johns Hopkins University	101
2	Volek Jeff	University System of Ohio	69
3	Rho Jong M.	University of Calgary	62
4	Yancy William S.	Duke University	45
5	Rodriguez Fernandez Maria Luz	University of Connecticut	43
6	Cross J. Helen	UCL Great Ormond St Inst Child and Lealth	43
7	Kim Heung Dong	Yonsei University Health System	40
8	Westman Eric	Lund University	39
9	Auvin Stéphane	University of California System	37
10	Clifton Peter Marshall	University of South Australia	37

### Analysis of journals

Researchers can accurately understand the core journals in a topic by analyzing its source journals, which also serves as a reliable resource for further field research (46). A total of 1,545 academic journals published 6,938 publications in the field of research on LChD from 2002 to 2021. As shown in Table 5, the top 10 journals accounted for 17.93% of the total publications. The most productive journals were Nutrients (292 publications), Epilepsia (203 publications), Epilepsy Research (134 publications), PLoS One (116 publications), and American Journal of Clinical Nutrition (105 publications). Of the top 10 journals, eight journals' IF more than 3.0. With a maximum of 8.472, the top 2 journals had an IF >6.0. This shows that high IF journals are open to publishing LChD research.

**Table 5:** The top 10 journals on LChD from 2002 to 2021.

**Rank**	**Journal**	**Publications**	**IF (2021)**
1	Nutrients	292	6.706
2	Epilepsia	203	6.740
3	Epilepsy Research	134	2.991
4	PLoS One	116	3.752
5	American Journal of Clinical Nutrition	105	8.472
6	Epilepsy and Behavior	96	3.337
7	Journal of Child Neurology	78	2.363
8	British Journal of Nutrition	76	4.125
9	Seizure European Journal of Epilepsy	74	3.414
10	Nutrition	73	4.893

**Figure 6:**
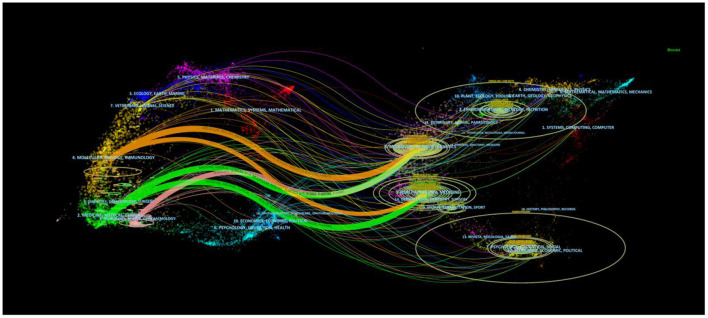
Dual-map overlay of academic journals from 2002 to 2021.

Figure 6 illustrated the dual- map overlay of journals that produced literature linked to the topic of LChD. On the map, the right labels represented the disciplines of the journals that published the cited papers, while the left labels represented the fields of the citing journals. Citation links can show the in and out of the citation dataset. Figure 6 showed 5 reference pathways. Three yellow pathways indicate articles published in molecular/biological/immunology journals mainly citing journals in the molecular/biological/genetics field. Two green pathways suggest that articles published in medicine/clinical journals mainly cite journals in the molecular/biology/genetics/health/nursing/medicine fields. One red pathway shows that the publications from neurology/sports/ophthalmology mainly cite journals in the in molecular/biology/genetics field.

### Analysis of co-cited references

**Figure 7:**
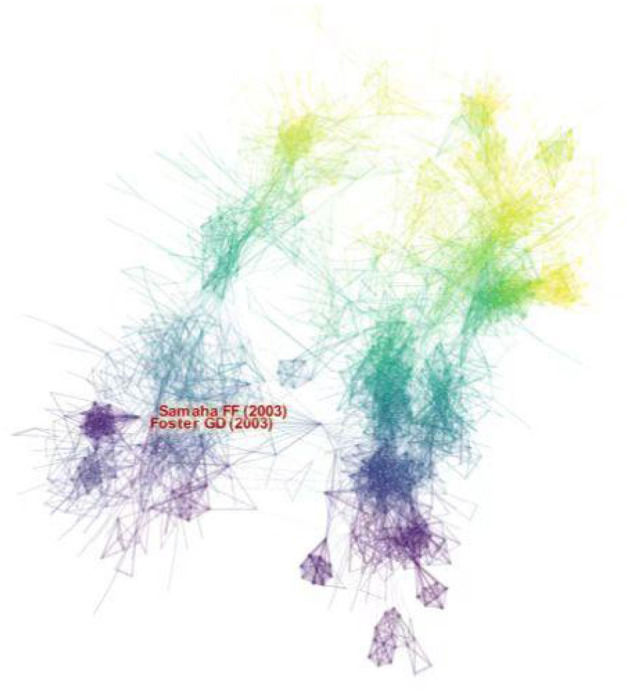
The map of co-cited reference on LChD from 2002 to 2021.

The co-cited reference analysis is one of the important indicators in bibliometric research and is usually used to explore research priorities in specific academic fields (47). CiteSpace generated the co-cited reference map, and 1,693 nodes and 9,455 links were shown in the map (Figure 7). The top 5 co-cited references in terms of frequency were in Table 6. Analysis of co-cited references provided basic data for LChD research. Noteworthy were three publications from the New England Journal of Medicine and two from the Annals of Internal Medicine, both of which have significant academic influence. The five references were all clinical trials. In most co-cited reference, obese people were given Atkins diet, and lost more weight in the first 6 months (48). Additionally, high density lipoprotein cholesterol levels increased and triglyceride levels decreased more in Atkins diet participants than in control group, indicating that Atkins diet had a higher impact on the risk factors for coronary heart disease. The second-most co-cited reference reported that patients who received a carbohydrate-restricted diet with 30 g per day or less, lost more weight than control group did and had relative improvements in their insulin sensitivity and triglyceride levels (49). The third most co-cited reference of 132 obese people on who were restricted carbohydrate intake to < 30 g per day showed more beneficial effects than those on conventional diets at 1 year; the effects of restricted carbohydrate on atherogenic dyslipidemia and glycemic control remained more favorable (34). Diet therapies were given to moderately obese subjects, and a low-carbohydrate diet and a Mediterranean diet were found to have beneficial effects on lipids and blood glucose, respectively (50). Individualized dietary regimens tailored to individual preferences and metabolism are recommended. A low-carbohydrate, ketogenic diet exhibited higher participant retention and more weight loss compared to low-fat diets in the literature with the sixth greatest co-citation frequency (51).

**Table 6:** The top 5 co-cited reference on LChD from 2002 to 2021.

**Rank**	**Frequency**	**Cited reference**	**Source**	**Reference**
1	287	A randomized trial of a low-carbohydrate diet for obesity	New England Journal of Medicine	(48)
2	234	A low-carbohydrate as compared with a low-fat diet in severe obesity	New England Journal of Medicine	(49)
3	178	The effects of low-carbohydrate vs. conventional weight loss diets in severely obese adults: 1-year follow-up of a randomized trial	Annals of Internal Medicine	(34)
4	171	Weight loss with a low-carbohydrate, Mediterranean, or low-fat diet	New England Journal of Medicine	(50)
5	154	A low-carbohydrate, ketogenic diet vs. a low-fat diet to treat obesity and hyperlipidemia: a randomized, controlled trial.	Annals of Internal Medicine	(51)

### References analysis

High cited references lay the foundation and accelerate the development of research in the field (23). The top 10 cited references were listed in Table 7. Of the top 10 references, 7 references were articles and 3 were reviews. Three references were published in the New England Journal of Medicine and two were published in Lancet. “Obesity” published by Haslam et al. in 2005, was cited 3,136 times, and ranked first. Shai et al. published in 2008 in New England Journal of Medicine of “Weight loss with a low-carbohydrate, Mediterranean, or low-fat diet” was cited 1,250 times, and ranked second. Foster et al. published in 2003 in “A randomized trial of a low-carbohydrate diet for obesity” in New England Journal of Medicinewas cited 1,124 times, and ranked third.

**Table 7:** The top 10 cited references on LChD from 2002 to 2021.

**Rank**	**Title**	**Author**	**Type**	**Journal**	**Year**	**Citations**
1	Obesity	Haslam DW, et al.	Review	*Lancet*	2005	3,136
2	Weight loss with a low-carbohydrate, Mediterranean, or low-fat diet	Shai I, et al.	Article	New England Journal of Medicine	2008	1,250
3	Hepatic fibroblast growth factor 21 is regulated by PPAR alpha and is a key mediator of hepatic lipid metabolism in ketotic states	Michael K Badman, et al.	Article	Cell Metabolism	2007	1,125
4	A randomized trial of a low-carbohydrate diet for obesity	Foster GD, et al.	Article	New England Journal of Medicine	2003	1,124
5	Comparison of the Atkins, Ornish, Weight watchers, and Zone diets for weight loss and heart disease risk reduction	Dansinger ML, et al.	Article	JAMA	2005	1,100
6	Nutrition recommendations and interventions for diabetes—a position statement of the American Diabetes Association	American Diabetes Association, et al.	Article	Diabetes Care	2008	1,074
7	Childhood obesity	Han JC, et al.	Review	Lancet	2010	1,010
8	Weight-loss outcomes: A systematic review and meta-analysis of weight-loss clinical trials with a minimum 1-year follow-up	Franz MJ, et al.	Review	Journal of the American Dietetic Association	2007	953
9	The ketone metabolite beta-hydroxybutyrate blocks NLRP3 inflammasome-mediated inflammatory disease	Youm YH, et al.	Article	Nature Medicine	2015	935
10	A low-carbohydrate as compared with a low-fat diet in severe obesity	Samaha FF, et al.	Article	New England Journal of Medicine	2003	844

### Analysis of keywords

**Figure 8:**
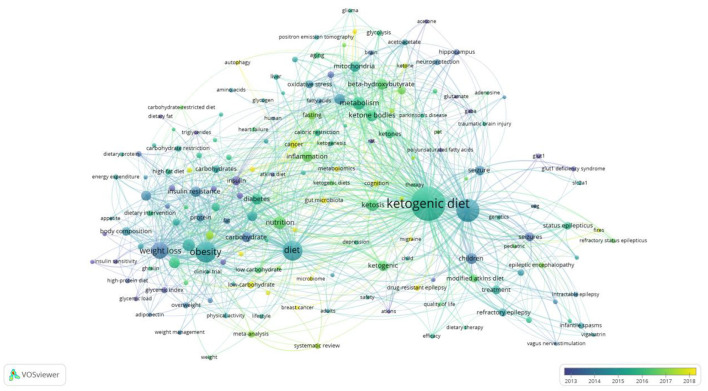
Map of keyword co-occurrence of overlay visualization on LChD from 2002 to 2021.

**Figure 9:**
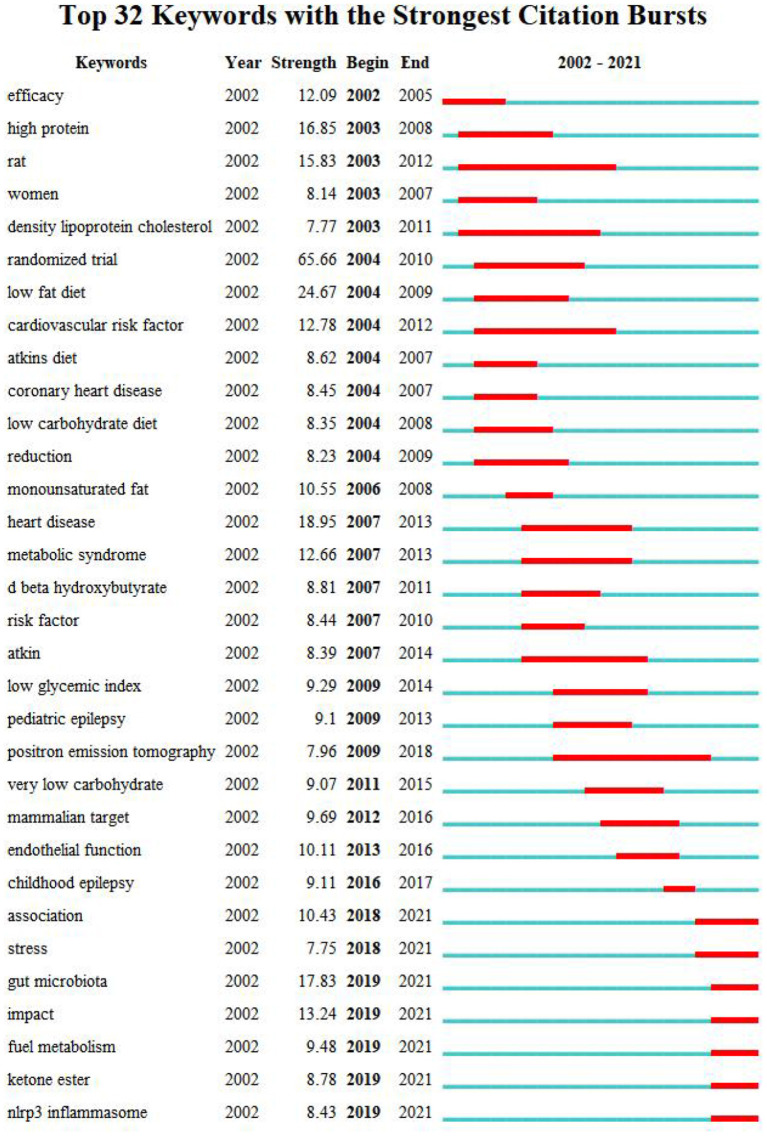
Map of keyword with the strongest citation bursts on LChD from 2002 to 2021.

The map of keywords can present the main research objects and the hot topics and frontiers trends. In this study, VOSviewer software performed the keyword co-occurrence of overlay visualization (Figure 8). A total of 9,750 keywords, 172 keywords met the thresholds when the minimum number of occurrences of a keywords was 20. From Figure 8, we found that the keywords research hotspots were categorized into “ketogenic diet,” “metabolism disease,” “cardiovascular disease” and “cancer.” Bursts keywords were frequently used at a period time, reflecting the frontiers trends. We used CiteSpace software to map the top 32 keywords with the strongest citation bursts from 2002 to 2021 (Figure 9). We summarized that “oxidative stress,” “gut microbiota,” and “inflammation factors” are becoming frontiers trends of LChD research in the future.

## Discussion

We performed a bibliometric analysis of the publications from WoSCC on LChD from 2002 to 2021 using CiteSpace and VOSviewer software. We then summarized the current status, hotspots and frontiers trends in this field.

A total of 6,938 publications including 5,350 articles and 1,588 reviews, related to LChD from 2002 to 2021 were retrieved by searching WOSCC database. The number of annual publications on LChD showed an overall upward trend in spite of fluctuation slightly in some years. LChD research mainly involved the categories of nutrition, endocrinology, and neurosciences, reflecting the multidisciplinary nature and comprehensive knowledge about LChD research. USA was with the largest number and the world science center in LChD field, and Australia, Canada, England, France and Germany maintained close cooperation relationships. Universities were major institutions for LChD research. Five of the top 10 institutions were from USA, which further confirmed US predominance in the field of LChD research. Duke University, Harvard University, Johns Hopkins University and University of Toronto had close collaboration relationships. The most productive authors were Eric Heath Kossoff, Jeff Scott Volek, Jong M. Rho, William S. Yancy, and Maria Luz Fernandez. The authors' collaboration showed a geographical concentration and general decentralization. The most productive journals were Nutrients, Epilepsia, Epilepsy Research, PLoS One, and American Journal of Clinical Nutrition. “A randomized trial of a low-carbohydrate diet for obesity” and “Obesity” were considered to be the most co-cited and cited reference respectively.

Based on the keywords the keyword co-occurrence of overlay visualization, we can explore the hotspots. From Figure 8, we summarized and analyzed four hotspots in LChD field. Here, we further analyzed the following aspects according to the application field of LChD: ketogenic diet, metabolism disease, cardiovascular disease and cancer.

- Ketogenic diet: The KD is a type of low-carb diet, characterized by high fat and very low carbohydrate. KD works on the basis of the biological principle of starvation, using fat as the main energy source of the body (52). KD initially achieved satisfactory results in pediatric refractory epilepsy and obesity (53). In recent years, research on KD has been extended to metabolic (54), cardiovascular (55), cancer (56), neurological (57), and respiratory (58) diseases with positive results, especially in hyperglycemia (59), hyperlipidemia (60), and insulin resistance (61). Of course there are some shortcomings. The long-term efficacy, such as weight regain (62), cardiovascular events (63), and bone metabolism (64), cannot be ignored. Therefore, the use of KD for disease treatment needs to be individualized according to different diseases and patients' data.
- Metabolism disease: In this study, obesity and diabetes are common metabolic diseases. By consuming less carbohydrates, restricting the body's usage of exogenous glucose, and boosting lipolysis and fatty acid oxidation to meet the body's energy needs, low carbohydrate nutrition enable individuals to lose weight (65). The studies have shown that LChD are efficient in assisting obese individuals reduce their weight (16, 66). In addition, a low-carbohydrate-high-fat diet can reduce the risk factors for obesity-related diseases while improving obesity and have good prospects for healthy weight loss (67). According to type 2 diabetes epidemiological report, a rise in carbohydrates is largely responsible for the increased calories in patients. Glycemic management begins with dietary carbohydrate restriction (68). One study has shown that patients who have dietary carbohydrate restriction maintains lower levels of glycosylated hemoglobin after a year (69). Research already have proven to the positive effects of LChD on the metabolic diseases of diabetes, obesity, and hypertension. Along with the changes in lifestyle and work style currently occurring, the current research on dietary nutrition and metabolism diseases is going to become a popular topic.
- Cardiovascular disease: The risk of cardiovascular disease rises with a high carbohydrate diet (70). The risk of cardiovascular disease can be decreased by a low carbohydrate nutrition (71). Blood pressure and cardiovascular disease morbidity and mortality are known to be strongly causally correlated. Several studies have shown that LChD can improve blood pressure by lowering diastolic and systolic pressure (72, 73). Increased levels of triglycerides, total cholesterol, and low-density lipoprotein cholesterol are crucial contributors to the development of atherosclerotic cardiovascular disease. Excessive levels of high-density lipoprotein cholesterol have a preventive impact, but elevated levels of total cholesterol and triglycerides are significant risk factors for atherosclerotic cardiovascular disease. A number of lipids, including triacylglycerol, total cholesterol, low density lipoprotein cholesterol, and high density lipoprotein cholesterol, are improved by LChD (74, 75). A reasonable LChD program is good for cardiovascular health since it has a long-term positive impact on the prevention of cardiovascular disease.
- Cancer: Nutrition is receiving increasing attention in oncology clinical research (76). Proper diet can prevent and treat cancer and reduce the incidence of cancer (77). Seyfried et al. (78) found that the rate of tumor growth is directly proportional to blood sugar levels. Reducing carbohydrate intake, especially KD, can make blood sugar at a low level and effectively inhibit tumor cell proliferation (79). Low-carb diet and KD can improve the quality of life, physical performance, body composition and metabolic health of cancer patients (80). Low-carb diet and KD may create an unfavorable metabolic environment for cancer cells. Therefore, LChD or/and standard therapy, enhance the potential of anti-tumor effects and improve quality of life (11).

Burst keywords can explore the future development trends. Therefore, we summarized the burst keywords into three aspects, and considered them to be frontiers trends of LChD field and anticipated to occur frequently in the future years.

- Oxidative stress: Oxidative stress is a negative effect produced by free radicals in the body, and it is considered to be an important pathogenic factor, such as diabetes mellitus, obesity, heart disease, and cancer (81). The metabolite of the carbohydrate is glucose. The intake of excessive carbohydrates produces more glucose and increases the oxidative pressure on mitochondria, which increases the production of excessive reactive oxygen species, leading to the occurrence of disease (82). Low-carbohydrate intake reduces to reduce the occurrence of oxidative stress in the body, thus reducing the incidence of disease (83). A review showed that the low-carbon ketogenic diet-mediated reduction in glucose levels and enhanced electron transport in the mitochondria further disrupt the energy metabolism of tumor cells, thus adversely affecting tumor cell proliferation (83).
- Gut microbiota: Gut flora can regulate body metabolism and participate in the occurrence of diseases through a variety of mechanisms. With increasing research on gut microbiota, the dietary pattern was identified as one of the main drivers of gut microbiota change. Recently, carbon aquatic ketone diet has been shown to effectively treat neurological diseases (84), tumor (85, 86), metabolic diseases (87), inflammatory bowel disease (88), etc. Its effect source is related to the participation of intestinal flora in neurodevelopment (84), various pathways to hinder tumor cell growth (89, 90), inhibit the growth of bifidobacterium and reduce the inflammatory factor (89). The current research on intestinal microbiota has explained the action mechanism of low-carbon ketogenic diet to some extent, but the number of studies is too small, requiring further exploration in the future to provide a more solid theoretical basis for the application of low-carbon ketogenic diet.
- Inflammation factors: With the development of life science and technology, the current literature also further explains the disease treatment and prevention of LChD from the inflammation level. Tumor necrosis factor-a, interleukin-6, lipocalin, and C-reactive protein are inflammation factors produced by adipose tissue (91– 94). Dietary habits can influence immune function and have anti-inflammatory effects (95). The diets of 9.6% energy from carbohydrate (96) and carbohydrate < 40 g/day (97) enhanced lipocalin and lowered C-reactive protein levels in obese patients. According to Jonasson et al. (98), type 2 diabetic individuals who took LChD of 20% energy from carbohydrate, had lower serum levels of interleukin-1 receptor and interleukin-6. An further experiment revealed that LChD lessens lipid deposition, avoids the buildup of plasma and aortic oxidation, lowers inflammatory cytokines in the arterial wall, and inhibits atherosclerosis (99). There will be a spectrum of levels at which research on LChD is conducted, with more inflammation factors becoming increasingly prevalent in the future.

## Limitations

To the best of our knowledge, the present study is the first bibliometric analysis to assess LChD. However, it has many limitations. First, considering that the data difference and incompleteness of other database data, we only analyzed publications from the WoSCC. Next, to better present the analysis result and to ensure the quality of the included literature, we included only articles and reviews published in English. This may lead to some screening bias.

## Conclusion

We searched all research publications related to LChD on the Web of Scientific Core Collection (WoSCC). CiteSpace software was used to analyze countries/regions, institutions, journals, authors, references, and keywords. LChD is a popular diet, attracting attention from scholars. The hotspots of LChD are three aspects, “metabolism disease,” “cardiovascular disease,” and “risk factor.” We summarize that “research on prevention and treatment,” “research on diet,” and “research on molecular level” are becoming frontiers trends of LChD research in the future directions and deserve further study.

## Data availability statement

The original contributions presented in the study are included in the article/supplementary material, further inquiries can be directed to the corresponding author.

## Author contributions

HP and GL: conceptualization. XH: methodology, writing-original draft preparation, and writing-review and editing. CL and LZ: software. LZ: investigation, data curation, and supervision. GL and XH: resources. GL and CL: visualization. All authors have read and agreed to the published version of the manuscript.

## References

[1] Rinonapoli G Pace V Ruggiero C Ceccarini P Bisaccia M Meccariello L. Obesity and bone: a complex relationship. Int J Mol Sci. (2021) 22:13662. 10.3390/ijms222413662 34948466 PMC8706946

[2] Gower BA Goss AM. A lower-carbohydrate, higher-fat diet reduces abdominal and intermuscular fat and increases insulin sensitivity in adults at risk of type 2 diabetes. J Nutr. (2015) 145:177S–83S. 10.3945/jn.114.195065 25527677 PMC4264021

[3] Accurso A Bernstein RK Dahlqvist A Draznin B Feinman RD Fine EJ. Dietary carbohydrate restriction in type 2 diabetes mellitus and metabolic syndrome: time for a critical appraisal. Nutr Metab. (2008) 5:9. 10.1186/1743-7075-5-9 18397522 PMC2359752

[4] York LW Puthalapattu S Wu GY. Nonalcoholic fatty liver disease and low-carbohydrate diets. Annu Rev Nutr. (2009) 29:365–79. 10.1146/annurev-nutr-070208-114232 19575599

[5] Diamond DM Alabdulgader AA de Lorgeril M Harcombe Z Kendrick M Malhotra A. Dietary recommendations for familial hypercholesterolaemia: an evidence-free zone. BMJ Evid Based Med. (2021) 6:295–301. 10.1136/bmjebm-2020-111412 32631832 PMC8639944

[6] Astrup A Meinert Larsen T Harper A. Atkins and other low-carbohydrate diets: hoax or an effective tool for weight loss? Lancet. (2004) 9437:897–9. 10.1016/S0140-6736(04)16986-9 15351198

[7] Kossoff EH Dorward JL. The modified Atkins diet. Epilepsia. (2008) 49:37–41. 10.1111/j.1528-1167.2008.01831.x 19049584

[8] Atallah R Filion KB Wakil SM Genest J Joseph L Poirier P. Long-term effects of 4 popular diets on weight loss and cardiovascular risk factors: a systematic review of randomized controlled trials. Circ Cardiovasc Qual Outcomes. (2014) 7:815–27. 10.1161/CIRCOUTCOMES.113.000723 25387778

[9] Muscogiuri G Barrea L Laudisio D Pugliese G Salzano C Savastano S. The management of very low-calorie ketogenic diet in obesity outpatient clinic: a practical guide. J Transl Med. (2019) 1:356. 10.1186/s12967-019-2104-z 31665015 PMC6820992

[10] Adam-Perrot A Clifton P Brouns F. Low-carbohydrate diets: nutritional and physiological aspects. Obes Rev. (2006) 7:49–58. 10.1111/j.1467-789X.2006.00222.x 16436102

[11] Weber DD Aminzadeh-Gohari S Tulipan J Catalano L Feichtinger RG Kofler B. Ketogenic diet in the treatment of cancer: where do we stand? Mol Metab. (2020) 33:102–21. 10.1016/j.molmet.2019.06.026 31399389 PMC7056920

[12] Paoli A Mancin L Bianco A Thomas E Mota JF Piccini F. Ketogenic diet and microbiota: friends or enemies? Genes. (2019) 10:534. 10.3390/genes10070534 31311141 PMC6678592

[13] Jovanovski E Zurbau A Vuksan V. Carbohydrates and endothelial function: is a low-carbohydrate diet or a low-glycemic index diet favorable for vascular health? Clin Nutr Res. (2015) 4:69–75. 10.7762/cnr.2015.4.2.69 25954727 PMC4418418

[14] Burke LM Sharma AP Heikura IA Forbes SF Holloway M McKay AK. Crisis of confidence averted: Impairment of exercise economy and performance in elite race walkers by ketogenic low carbohydrate, high fat (LCHF) diet is reproducible. PLoS ONE. (2020) 6:e0234027. 10.1371/journal.pone.0234027 32497061 PMC7272074

[15] Huntriss R Campbell M Bedwell C. The interpretation and effect of a low-carbohydrate diet in the management of type 2 diabetes: a systematic review and meta-analysis of randomised controlled trials. Eur J Clin Nutr. (2018) 72:311–25. 10.1038/s41430-017-0019-4 29269890

[16] Ebbeling CB Feldman HA Klein GL Wong JM Bielak L Steltz SK. Effects of a low carbohydrate diet on energy expenditure during weight loss maintenance: randomized trial [published correction appears in BMJ. 2020 Nov 3;371:m4264]. BMJ. (2018) 363:k4583. 10.1136/bmj.k4583 30429127 PMC6233655

[17] Hite AH Berkowitz VG Berkowitz K. Low-carbohydrate diet review: shifting the paradigm. Nutr Clin Pract. (2011) 26:300–8. 10.1177/0884533611405791 21586415

[18] Oelrich B Peters R Jung K. A bibliometric evaluation of publications in urological journals among European Union countries between 2000 and 2005. Eur Urol. (2007) 52:1238–48. 10.1016/j.eururo.2007.06.050 17673361

[19] Aydinoglu AU Taşkin Z. Origins of life research: a bibliometric approach. Orig Life Evol Biosph. (2018) 48:55–71. 10.1007/s11084-017-9543-4 28702783

[20] Galetsi P Katsaliaki K. Big data analytics in health: an overview and bibliometric study of research activity. Health Info Libr J. (2020) 37:5–25. 10.1111/hir.12286 31889380

[21] Blake H Bermingham F Johnson G Tabner A. Mitigating the psychological impact of COVID-19 on healthcare workers: a digital learning package. Int J Environ Res Public Health. (2020) 9:2997. 10.3390/ijerph17092997 32357424 PMC7246821

[22] Li W Weng L Xiang Q Fan T. Trends in research on traditional Chinese health exercises for improving cognitive function: a bibliometric analysis of the literature from 2001 to 2020. Front Public Health. (2022) 9:794836. 10.3389/fpubh.2021.794836 35071171 PMC8770942

[23] You Y Li W Liu J Li X Fu Y Ma X. Bibliometric review to explore emerging high-intensity interval training in health promotion: a new century picture. Front Public Health. (2021) 9:697633. 10.3389/fpubh.2021.697633 34368063 PMC8342813

[24] Lu C Li X Yang K. Trends in shared decision-making studies from 2009 to 2018: a bibliometric analysis. Front Public Health. (2019) 7:384. 10.3389/fpubh.2019.00384 31921749 PMC6930165

[25] Waqas A Teoh SH Lapão LV Messina LA Correia JC. Harnessing telemedicine for the provision of health care: bibliometric and scientometric analysis. J Med Internet Res. (2020) 22:e18835. 10.2196/18835 33006571 PMC7568215

[26] Zheng KY Dai GY Lan Y Wang XQ. Trends of repetitive transcranial magnetic stimulation from 2009 to 2018: a bibliometric analysis. Front Neurosci. (2020) 14:106. 10.3389/fnins.2020.00106 32174808 PMC7057247

[27] Wang XQ Peng MS Weng LM Zheng YL Zhang ZJ Chen PJ. Bibliometric study of the comorbidity of pain and depression research. Neural Plast. (2019) 2019:1657498. 10.1155/2019/1657498 31772566 PMC6854239

[28] Murdayanti Y Khan MNAA. The development of internet financial reporting publications: a concise of bibliometric analysis. Heliyon. (2021) 12:e08551. 10.1016/j.heliyon.2021.e08551 34934847 PMC8654794

[29] Leal Filho W Ternova L Parasnis SA Kovaleva M Nagy GJ. Climate change and zoonoses: a review of concepts, definitions, and bibliometrics. Int J Environ Res Public Health. (2022) 2:893. 10.3390/ijerph19020893 35055715 PMC8776135

[30] Qi Q. The retrieval and application of web of science. Library Work Study. (2013) 2:110–2.

[31] Pulford DS. Ketogenic diets for epileptics. Cal West Med. (1927) 1:50–6. PMC1655541 18740398

[32] Pankey LD. Control of dental caries by a low carbohydrate diet. J Fla State Dent Soc. (1948) 19:5–7. 18889992

[33] Stern L Iqbal N Seshadri P Chicano KL Daily DA McGrory J. The effects of low-carbohydrate vs. conventional weight loss diets in severely obese adults: 1-year follow-up of a randomized trial. Ann Intern Med. (2004) 140:778–85. 10.7326/0003-4819-140-10-200405180-00007 15148064

[34] Kamuren ZT Sanders R Watkins JB. Low-carbohydrate diet and oxidative stress in diabetic and nondiabetic rats. J Biochem Mol Toxicol. (2006) 20:259–69. 10.1002/jbt.20142 17009256

[35] Focardi M Dick GM Picchi A Zhang C Chilian WM. Restoration of coronary endothelial function in obese Zucker rats by a low-carbohydrate diet. Am J Physiol Heart Circ Physiol. (2007) 292:H2093–9. 10.1152/ajpheart.01202.2006 17220180

[36] Shi GJ Geng Y. Science is flat: new explanation of Yuasa phenomenon. Stud Sci Educ. (2012) 30:1770–80.

[37] Dang Q Luo Z Ouyang C Wang L. First systematic review on health communication using the CiteSpace software in China: exploring its research hotspots and frontiers. Int J Environ Res Public Health. (2021) 18:13008. 10.3390/ijerph182413008 34948617 PMC8702194

[38] McDonald TJW Ratchford EV Henry-Barron BJ Kossoff EH Cervenka MC. Impact of the modified Atkins diet on cardiovascular health in adults with epilepsy. Epilepsy Behav. (2018) 79:82–6. 10.1016/j.yebeh.2017.10.035 29253679

[39] Cervenka MC Patton K Eloyan A Henry B Kossoff EH. The impact of the modified Atkins diet on lipid profiles in adults with epilepsy. Nutr Neurosci. (2016) 3:131–7. 10.1179/1476830514Y.0000000162 25383724

[40] deCampo DM Kossoff EH. Ketogenic dietary therapies for epilepsy and beyond. Curr Opin Clin Nutr Metab Care. (2019) 4:264–8. 10.1097/MCO.0000000000000565 31033577

[41] Volek JS Ballard KD Silvestre R Judelson DA Quann EE Forsythe CE. Effects of dietary carbohydrate restriction vs. low-fat diet on flow-mediated dilation. Metabolism. (2009) 58:1769–77. 10.1016/j.metabol.2009.06.005 19632695

[42] Leite JO DeOgburn R Ratliff J Su R Smyth JA Volek JS. Low-carbohydrate diets reduce lipid accumulation and arterial inflammation in guinea pigs fed a high-cholesterol diet. Atherosclerosis. (2010) 209:442–8. 10.1016/j.atherosclerosis.2009.10.005 19892353

[43] Chun KC Ma SC Oh H Rho JM Kim DY. Ketogenic diet-induced extension of longevity in epileptic Kcna1-null mice is influenced by gender and age at treatment onset. Epilepsy Res. (2018) 140:53–5. 10.1016/j.eplepsyres.2017.11.005 29245026 PMC5826793

[44] Cheng N Rho JM Masino SA. A metabolic dysfunction underlying autism spectrum disorder and potential treatment approaches. Front Mol Neurosci. (2017) 10:34. 10.3389/fnmol.2017.00034 28270747 PMC5318388

[45] Ahn Y Sabouny R Villa BR Yee NC Mychasiuk R Uddin GM. Aberrant mitochondrial morphology and function in the BTBR mouse model of autism is improved by 2 weeks of ketogenic diet. Int J Mol Sci. (2020) 9:3266. 10.3390/ijms21093266 32380723 PMC7246481

[46] Shi D Xie C Wang J Xiong L. Changes in the structures and directions of heavy metal-contaminated soil remediation research from 1999 to 2020: a bibliometric and scientometric study. Int J Environ Res Public Health. (2021) 18:7358. 10.3390/ijerph18147358 34299808 PMC8303952

[47] Trujillo CM Long TM. Document co-citation analysis to enhance transdisciplinary research. Sci Adv. (2018) 4:e1701130. 10.1126/sciadv.1701130 29308433 PMC5752411

[48] Foster GD Wyatt HR Hill JO McGuckin BG Brill C Mohammed BS. A randomized trial of a low-carbohydrate diet for obesity. N Engl J Med. (2003) 348:2082–90. 10.1056/NEJMoa022207 12761365

[49] Samaha FF Iqbal N Seshadri P Chicano KL Daily DA McGrory J. A low-carbohydrate as compared with a low-fat diet in severe obesity. N Engl J Med. (2003) 348:2074–81. 10.1056/NEJMoa022637 12761364

[50] Shai I Schwarzfuchs D Henkin Y Shahar DR Witkow S Greenberg I. Weight loss with a low-carbohydrate, Mediterranean, or low-fat diet [published correction appears in N Engl J Med. 2009 Dec 31;361(27):2681]. N Engl J Med. (2008) 359:229–41. 10.1056/NEJMoa0708681 18635428

[51] Yancy WS Olsen MK Guyton JR Bakst RP Westman EC A. low-carbohydrate, ketogenic diet vs. a low-fat diet to treat obesity and hyperlipidemia: a randomized, controlled trial. Ann Intern Med. (2004) 140:769–77. 10.7326/0003-4819-140-10-200405180-00006 15148063

[52] Dhamija R Eckert S Wirrell E. Ketogenic diet. Can J Neurol Sci. (2013) 2:158–67. 10.1017/S0317167100013676 23419562

[53] Vasquez A Farias-Moeller R Sánchez-Fernández I Abend NS Amengual-Gual M Anderson A. Super-refractory status epilepticus in children: a retrospective cohort study. Pediatr Crit Care Med. (2021) 12:e613–25. 10.1097/PCC.0000000000002786 34120133

[54] Gangitano E Gnessi L Lenzi A Ray D. Chronobiology and metabolism: is ketogenic diet able to influence circadian rhythm? Front Neurosci. (2021) 15:756970. 10.3389/fnins.2021.756970 34819833 PMC8606558

[55] Tzenios N Lewis ED Crowley DC Chahine M Evans M. Examining the efficacy of a very-low-carbohydrate ketogenic diet on cardiovascular health in adults with mildly elevated low-density lipoprotein cholesterol in an open-label pilot study. Metab Syndr Relat Disord. (2022) 2:94–103. 10.1089/met.2021.0042 34918971 PMC8972001

[56] Shen W He J Hou T Si J Chen S. Common pathogenetic mechanisms underlying aging and tumor and means of interventions. Aging Dis. (2022) 4:1063–91. 10.14336/AD.2021.1208 35855334 PMC9286910

[57] Xu Y Jiang C Wu J Deng X Zhang Y Peng B. Ketogenic diet ameliorates cognitive impairment and neuroinflammation in a mouse model of Alzheimer's disease. CNS Neurosci Ther. (2022) 4:580–92. 10.1111/cns.13779 34889516 PMC8928920

[58] Melkonian SC Daniel CR Ye Y Pierzynski JA Roth JA Wu X. Glycemic index, glycemic load, and lung cancer risk in non-hispanic whites. Cancer Epidemiol Biomarkers Prev. (2016) 3:532–9. 10.1158/1055-9965.EPI-15-0765 26944871 PMC4780226

[59] Montemurro N Perrini P Rapone B. Clinical risk and overall survival in patients with diabetes mellitus, hyperglycemia and glioblastoma multiforme. A review of the current literature. Int J Environ Res Public Health. (2020) 22:8501. 10.3390/ijerph17228501 33212778 PMC7698156

[60] Hall KD Chung ST. Low-carbohydrate diets for the treatment of obesity and type 2 diabetes. Curr Opin Clin Nutr Metab Care. (2018) 4:308–12. 10.1097/MCO.0000000000000470 29677013

[61] Buehler LA Noe D Knapp S Isaacs D Pantalone KM. Ketogenic diets in the management of type 1 diabetes: safe or safety concern? Cleve Clin J Med. (2021) 10:547–55. 10.3949/ccjm.88a.20121 34598919

[62] Deemer SE Plaisance EP Martins C. Impact of ketosis on appetite regulation: a review. Nutr Res. (2020) 77:1–11. 10.1016/j.nutres.2020.02.010 32193016

[63] Landry MJ Crimarco A Gardner CD. Benefits of low carbohydrate diets: a settled question or still controversial? Curr Obes Rep. (2021) 10:409–22. 10.1007/s13679-021-00451-z 34297345 PMC9621749

[64] Shu L Beier E Sheu T Zhang H Zuscik MJ Puzas EJ. High-fat diet causes bone loss in young mice by promoting osteoclastogenesis through alteration of the bone marrow environment. Calcif Tissue Int. (2015) 4:313–23. 10.1007/s00223-015-9954-z 25673503 PMC4383048

[65] Westman EC Feinman RD Mavropoulos JC Vernon MC Volek JS Wortman JA. Low carbohydrate nutrition and metabolism. Am J Clin Nutr. (2007) 86:276–84. 10.1093/ajcn/86.2.276 17684196

[66] Gardner CD Trepanowski JF Del Gobbo LC Hauser ME Rigdon J Ioannidis JP. Effect of low-fat vs. low-carbohydrate diet on 12-month weight loss in overweight adults and the association with genotype pattern or insulin secretion: the DIETFITS randomized clinical trial. JAMA. (2018) 319:667–79. 10.1001/jama.2018.0245 29466592 PMC5839290

[67] Brouns F. Overweight and diabetes prevention: is a low-carbohydrate-high-fat diet recommendable? Eur J Nutr. (2018) 57:1301–12. 10.1007/s00394-018-1636-y 29541907 PMC5959976

[68] Feinman RD Pogozelski WK Astrup A Bernstein RK Fine EJ Westman EC. Dietary carbohydrate restriction as the first approach in diabetes management: critical review and evidence base. Nutrition. (2015) 31:1–13. 10.1016/j.nut.2014.06.011 25287761

[69] Rock CL Flatt SW Pakiz B Taylor KS Leone AF Brelje K. Weight loss, glycemic control, and cardiovascular disease risk factors in response to differential diet composition in a weight loss program in type 2 diabetes: a randomized controlled trial. Diabet Care. (2014) 37:1573–80. 10.2337/dc13-2900 24760261 PMC4392939

[70] Asadi Z Shafiee M Sadabadi F Heidari-Bakavoli A Moohebati M Khorrami MS. Association of dietary patterns and risk of cardiovascular disease events in the MASHAD cohort study. J Hum Nutr Diet. (2019) 32:789–801. 10.1111/jhn.12669 31332855

[71] Das S McCreary J Shamim S Kalayjian T. Reversal of severe hypertriglyceridemia with intermittent fasting and a very-low-carbohydrate ketogenic diet: a case series. Curr Opin Endocrinol Diabetes Obes. (2020) 27:308–11. 10.1097/MED.0000000000000566 32740049

[72] Evans CE Greenwood DC Threapleton DE Cleghorn CL Nykjaer C Woodhead CE. Effects of dietary fibre type on blood pressure: a systematic review and meta-analysis of randomized controlled trials of healthy individuals. J Hypertens. (2015) 33:897–911. 10.1097/HJH.0000000000000515 25668347

[73] Flint AJ Hu FB Glynn RJ Jensen MK Franz M Sampson L. Whole grains and incident hypertension in men. Am J Clin Nutr. (2009) 90:493–8. 10.3945/ajcn.2009.27460 19571218 PMC2728640

[74] Hu T Bazzano LA. The low-carbohydrate diet and cardiovascular risk factors: evidence from epidemiologic studies. Nutr Metab Cardiovasc Dis. (2014) 24:337–43. 10.1016/j.numecd.2013.12.008 24613757 PMC4351995

[75] Kim JY Yang YH Kim CN Lee CE Kim KI. Effects of very-low-carbohydrate (horsemeat- or beef-based) diets and restricted feeding on weight gain, feed and energy efficiency, as well as serum levels of cholesterol, triacylglycerol, glucose, insulin and ketone bodies in adult rats. Ann Nutr Metab. (2008) 3–4:260–7. 10.1159/000189129 19136821

[76] Haskins C Cohen J Kotecha R Kaiser A. Low carbohydrate diets in cancer therapeutics: current evidence. Front Nutr. (2021) 8:662952. 10.3389/fnut.2021.662952 34901101 PMC8655114

[77] Ravasco P Monteiro-Grillo I Vidal PM Camilo ME. Nutritional deterioration in cancer: the role of disease and diet. Clin Oncol. (2003) 15:443–50. 10.1016/S0936-6555(03)00155-9 14689999

[78] Seyfried TN Marsh J Shelton LM Huysentruyt LC Mukherjee P. Is the restricted ketogenic diet a viable alternative to the standard of care for managing malignant brain cancer? Epilepsy Res. (2012) 3:310–26. 10.1016/j.eplepsyres.2011.06.017 21885251

[79] Champ CE Palmer JD Volek JS Werner-Wasik M Andrews DW Evans JJ. Targeting metabolism with a ketogenic diet during the treatment of glioblastoma multiforme. J Neurooncol. (2014) 117:125–31. 10.1007/s11060-014-1362-0 24442482

[80] Kämmerer U Klement RJ Joos FT Sütterlin M Reuss-Borst M. Low carb and ketogenic diets increase quality of life, physical performance, body composition, and metabolic health of women with breast cancer. Nutrients. (2021) 3:1029. 10.3390/nu13031029 33806775 PMC8004887

[81] Karam BS Chavez-Moreno A Koh W Akar JG Akar FG. Oxidative stress and inflammation as central mediators of atrial fibrillation in obesity and diabetes. Cardiovasc Diabetol. (2017) 1:120. 10.1186/s12933-017-0604-9 28962617 PMC5622555

[82] Sinha K Das J Pal PB Sil PC. Oxidative stress: the mitochondria-dependent and mitochondria-independent pathways of apoptosis. Arch Toxicol. (2013) 7:1157–80. 10.1007/s00204-013-1034-4 23543009

[83] Feng S Wang H Liu J Aa J Zhou F Wang G. Multi-dimensional roles of ketone bodies in cancer biology: opportunities for cancer therapy. Pharmacol Res. (2019) 150:104500. 10.1016/j.phrs.2019.104500 31629092

[84] Carlson AL Xia K Azcarate-Peril MA Goldman BD Ahn M Styner MA. Infant gut microbiome associated with cognitive development. Biol Psychiatry. (2018) 2:148–59. 10.1016/j.biopsych.2017.06.021 28793975 PMC5724966

[85] Dai X Bu X Gao Y Guo J Hu J Jiang C. Energy status dictates PD-L1 protein abundance and anti-tumor immunity to enable checkpoint blockade. Mol Cell. (2021) 11:2317–31.e6. 10.1016/j.molcel.2021.03.037 33909988 PMC8178223

[86] Ferrere G Alou MT Liu P Goubet AG Fidelle M Kepp O. Ketogenic diet and ketone bodies enhance the anticancer effects of PD-1 blockade. JCI Insight. (2021) 2:e145207. 10.1172/jci.insight.145207 33320838 PMC7934884

[87] Abbasi J. Interest in the ketogenic diet grows for weight loss and type 2 diabetes. JAMA. (2018) 3:215–7. 10.1001/jama.2017.20639 29340675

[88] Ang QY Alexander M Newman JC Tian Y Cai J Upadhyay V. Ketogenic diets alter the gut microbiome resulting in decreased intestinal Th17 cells. Cell. (2020) 6:1263–75.e16. 10.1016/j.cell.2020.04.027 32437658 PMC7293577

[89] Lane J Brown NI Williams S Plaisance EP Fontaine KR. Ketogenic diet for cancer: critical assessment and research recommendations. Nutrients. (2021) 10:3562. 10.3390/nu13103562 34684564 PMC8539953

[90] Mundi MS Mohamed Elfadil O Patel I Patel J Hurt RT. Ketogeniciet and cancer: fad or fabulous? JPEN J Parenter Enteral Nutr. (2021) S2:26–32. 10.1002/jpen.2226 34897736

[91] Barry JC Simtchouk S Durrer C Jung ME Little JP. Short-term exercise training alters leukocyte chemokine receptors in obese adults. Med Sci Sports Exerc. (2017) 49:1631–40. 10.1249/MSS.0000000000001261 28319586

[92] Pedersen BK Fischer CP. Beneficial health effects of exercise–the role of IL-6 as a myokine. Trends Pharmacol Sci. (2007) 28:152–6. 10.1016/j.tips.2007.02.002 17331593

[93] Galic S Oakhill JS Steinberg GR. Adipose tissue as an endocrine organ. Mol Cell Endocrinol. (2009) 6:129–39. 10.1016/j.mce.2009.08.018 19723556

[94] Ziemke F Mantzoros CS. Adiponectin in insulin resistance: lessons from translational research. Am J Clin Nutr. (2009) 10:258S–61S. 10.3945/ajcn.2009.28449C 19906806 PMC2793112

[95] Romanowska-Próchnicka K Felis-Giemza A Olesińska M Wojdasiewicz P Paradowska-Gorycka A Szukiewicz D. The role of TNF-α and Anti-TNF-α agents during preconception, pregnancy, and breastfeeding. Int J Mol Sci. (2021) 20:2922. 10.3390/ijms22062922 33805757 PMC7998738

[96] Ruth MR Port AM Shah M Bourland AC Istfan NW Nelson KP. Consuming a hypocaloric high fat low carbohydrate diet for 12 weeks lowers C-reactive protein, and raises serum adiponectin and high density lipoprotein-cholesterol in obese subjects. Metabolism. (2013) 62:1779–87. 10.1016/j.metabol.2013.07.006 24075505 PMC3845365

[97] Hu T Yao L Reynolds K Whelton PK Niu T Li S. The effects of a low-carbohydrate diet vs. a low-fat diet on novel cardiovascular risk factors: a randomized controlled trial. Nutrients. (2015) 7:7978–94. 10.3390/nu7095377 26393645 PMC4586572

[98] Jonasson L Guldbrand H Lundberg AK Nystrom FH. Advice to follow a low-carbohydrate diet has a favorable impact on low-grade inflammation in type 2 diabetes compared with advice to follow a low-fat diet. Ann Med. (2014) 46:182–7. 10.3109/07853890.2014.894286 24779961 PMC4025600

[99] Zhang W An R Li Q Sun L Lai X Chen R. Theaflavin TF3 relieves hepatocyte lipid deposition through activating an AMPK signaling pathway by targeting plasma kallikrein. J Agric Food Chem. (2020) 68:2673–83. 10.1021/acs.jafc.0c00148 32050765

